# Does a shift in host plants trigger speciation in the Alpine leaf beetle *Oreina speciosissima *(Coleoptera, Chrysomelidae)?

**DOI:** 10.1186/1471-2148-11-310

**Published:** 2011-10-20

**Authors:** Matthias Borer, Tom van Noort, Nils Arrigo, Sven Buerki, Nadir Alvarez

**Affiliations:** 1 Museum of Natural History Neuchâtel, Rue des Terreaux 14, 2000 Neuchâtel, Switzerland; 2 Laboratory of Evolutionary Entomology, Institute of Biology, University of Neuchâtel, Rue Emile-Argand 11, 2000 Neuchâtel, Switzerland; 3 Department of Ecology & Evolutionary Biology, University of Arizona, Tucson, AZ, 85721, USA; 4 Jodrell Laboratory, Royal Botanic Gardens, Kew, Richmond, Surrey TW9 3DS, UK; 5 Department of Ecology and Evolution, University of Lausanne, Biophore Building, 1015 Lausanne, Switzerland

## Abstract

**Background:**

Within the Coleoptera, the largest order in the animal kingdom, the exclusively herbivorous Chrysomelidae are recognized as one of the most species rich beetle families. The evolutionary processes that have fueled radiation into the more than thirty-five thousand currently recognized leaf beetle species remain partly unresolved. The prominent role of leaf beetles in the insect world, their omnipresence across all terrestrial biomes and their economic importance as common agricultural pest organisms make this family particularly interesting for studying the mechanisms that drive diversification. Here we specifically focus on two ecotypes of the alpine leaf beetle *Oreina speciosissima *(Scop.), which have been shown to exhibit morphological differences in male genitalia roughly corresponding to the subspecies *Oreina speciosissima sensu stricto *and *Oreina speciosissima troglodytes*. In general the two ecotypes segregate along an elevation gradient and by host plants: *Oreina speciosissima sensu stricto *colonizes high forb vegetation at low altitude and *Oreina speciosissima troglodytes *is found in stone run vegetation at higher elevations. Both host plants and leaf beetles have a patchy geographical distribution. Through use of gene sequencing and genome fingerprinting (AFLP) we analyzed the genetic structure and habitat use of *Oreina speciosissima *populations from the Swiss Alps to examine whether the two ecotypes have a genetic basis. By investigating a wide range of altitudes and focusing on the structuring effect of habitat types, we aim to provide answers regarding the factors that drive adaptive radiation in this phytophagous leaf beetle.

**Results:**

While little phylogenetic resolution was observed based on the sequencing of four DNA regions, the topology and clustering resulting from AFLP genotyping grouped specimens according to their habitat, mostly defined by plant associations. A few specimens with intermediate morphologies clustered with one of the two ecotypes or formed separate clusters consistent with habitat differences. These results were discussed in an ecological speciation framework.

**Conclusions:**

The question of whether this case of ecological differentiation occurred in sympatry or allopatry remains open. Still, the observed pattern points towards ongoing divergence between the two ecotypes which is likely driven by a recent shift in host plant use.

## Background

The debate about the relative importance of ecological speciation in species diversification spans several decades [1-20]. However, concrete cases based on empirical evidence remain relatively scarce [1,21-25]. In essence, ecological speciation is related to the "ecological species concept", which was defined as follows [26]: "a species is a lineage (or a closely related set of lineages), which occupies an adaptive zone minimally different from that of any other lineage in its range and which evolves separately from all lineages outside its range". The driving force behind ecological speciation is thus divergent natural selection between environments or, in other words, reproductive isolation of populations by means of adaptation to different environments or niches [18,19,21,27,28]. Ecological selection is a consequence of individual-based interactions with the environment. From this interaction follows that divergent selection between ecological niches is a major driving force differentiating lineages until reproductive isolation occurs [17]. Ecologically divergent pairs of populations will show higher levels of reproductive incompatibility and lower levels of gene flow than ecologically more similar population pairs [29]. A resulting corollary is that ecological speciation is more likely to arise in regions with patchworks of contrasting habitats and/or distinct environmental gradients.

The number of taxa within the insect order Coleoptera exceeds that of any known plant or animal group [30]. More than half of the beetles are phytophagous, including the species rich superfamilies Curculionoidea and Chrysomeloidea, of which a majority feeds on angiosperms [31]. The increase in phytophagous beetle diversity was facilitated by the rise of flowering plants [31]. The family Chrysomelidae currently consists of more than thirty-five thousand recognized species including economically important pest species such as the Colorado potato beetle (*Leptinotarsa decemlineata*), the Northern corn rootworm (*Diabrotica virgifera*), the Cereal leaf beetle (*Oulema melanopus*), and the Striped turnip flea beetle (*Phyllotreta nemorum*). The biological and economic importance of the superfamily Chrysomeloidea make it vital to understand the factors that drive diversification in this group.

Here, we present a case of ecological niche differentiation in the alpine leaf beetle *Oreina speciosissima *that may represent the early stages of ecological speciation. The genus *Oreina *currently includes twenty-eight species, of which only seven early-diverging taxa do not exclusively occur in high forbs (i.e. five develop in stone run vegetation and two can be found in both high forbs and stone runs) [32]. According to current knowledge [34], the most parsimonious explanation is that high forbs vegetation is the ancestral niche for the remaining twenty-one *Oreina *lineages, among which only our focal taxon *Oreina speciosissima *shows a partial reversal, since it is found both in high forbs and stone run vegetation.

**Figure 1:**
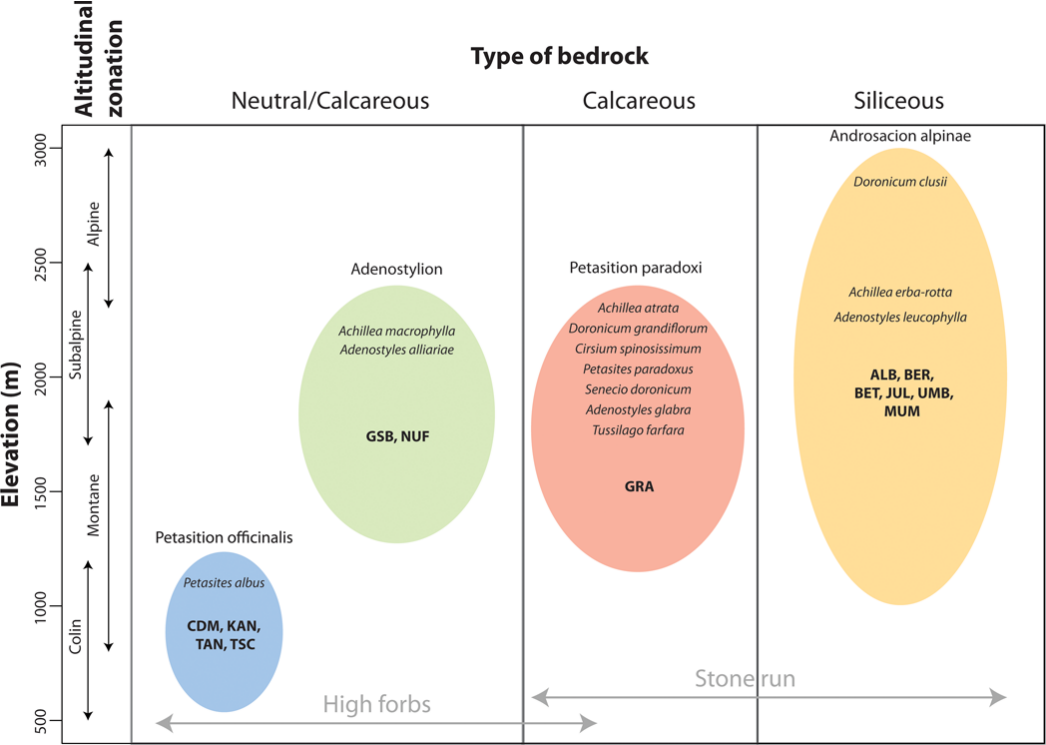
**Host plants of *Oreina speciosissima *and their altitudual zonation and habitat**. According to literature [32] and personal observations of the authors, 13 plant species were referred to as putative host plants for *Oreina speciosissima*. These species belong to four plant associations, i.e. Petasition officinalis, Adenostylion, Petasition paradoxi and Androsacion alpinae [33], which segregate along altitudinal zonation and bedrock type gradients. Whereas Petasition officinalis and Adenostyles correspond to high forbs habitat, Petasition paradoxi and Androsacion alpinae represent stone run habitats. In particular conditions, the Petasition paradoxi can merge with high forbs (see text). Each association includes bold written codes (see Table 1 for details) of sampled populations. Plant associations are written from left to right as a function of their mean elevation, and also according to a putative scenario of colonization of central Alpine silicious stone run vegetation by specific *Oreina speciosissima *lineages (high forbs representing the original habitat for most *Oreina *species). Main host plant species of *Oreina speciosissima *in each of the four associations are as follows: *Petasites albus *in Petasition officinalis, *Adenostyles alliariae *in Adenostylion, *Doronicum grandiflorum *in Petasition paradoxi, and *Doronicum clusii *in Androsacion alpinae.

*Oreina speciosissima *is distributed across nearly the entire range of the genus *Oreina *(from the Pyrenees in the west to the Carpathian Mountains in the east) through a wide altitudinal gradient (ranging from 800 to 2700 m above sea level). At lower elevations it generally colonizes the very abundant high forbs vegetation whereas at higher elevations it is found in stone run habitats across a small portion of its distribution range [unpublished observations MB, TVN][32]. Kippenberg [32] and personal observations suggest that *Oreina speciosissima *feeds exclusively on *Asteraceae *(*Achillea, Adenostyles, Cirsium, Doronicum, Petasites, Senecio *and *Tussilago*) and colonizes four distinct habitats represented by well-established plant associations: two occurring in high forbs - Petasition officinalis and Adenostylion - and two in stone run -Androsacion alpinae on siliceous bedrock and Petasition paradoxi on calcareous bedrock - (see Figure 1)[33]. These plant communities are often patchily distributed due to the myriad of spatially proximate microclimates that occur in the Alps, especially sites with calcareous bedrocks which regularly present a mosaic of microhabitats. For instance, sinkholes or dolines, formed through water erosion in so-called karstic areas represent ecological islands inhabited by high forbs vegetation surrounded by areas covered by stone run vegetation [unpublished observation TVN]. Beetles inhabiting the highly divergent habitats have been categorized in two different subspecies, namely *Oreina speciosissima sensu stricto *(distributed across the whole species range) and *Oreina speciosissima troglodytes *(restricted to the Swiss and neighbouring Italian Alps), on the basis of differences in elytral coloration and the shape of male genitalia (aedeagus) [32]. *Oreina speciosissima sensu stricto *beetles are bright metallic green or blue whereas the coloration in *Oreina speciosissima troglodytes *is generally darker and mat [32]. It is not known whether the morphological differences between ecotypes are in any way adaptive and/or have a genetic basis, although color patterns in another species of *Oreina *are known to influence natural selection through predation pressure [35]. Like most other members of the genus, *Oreina speciosissima sensu stricto *can be found feeding or mating throughout the day on, or in the vicinity of its host plants. In contrast, *Oreina speciosissima troglodytes *is usually found adjacent to its host plants, concealed in crevices and under loose rocks. Previous studies by the authors [36,37] greatly challenged the existence of clear species boundaries within the genus: it is therefore realistic from a biological point of view to refer to these taxonomic entities as ecotypes rather than subspecies. Leaf beetles from the genus *Oreina *are generally thought to make only limited use of their dispersal capabilities [38,39] even though Kalberer *et al*. [40] reported an average flight dispersal of approximately one hundred meters for *Oreina cacaliae *beetles during autumn migration. Rowell-Rahier [35] showed that low vagility in concert with a patchy host plant distribution resulted in a low level of genetic structuring in *Oreina speciosissima. Oreina speciosissima sensu stricto *beetles inhabit high forbs patches that are generally larger in size, lie closer together and harbor more beetles per unit of surface area than stone run patches inhabited by *Oreina speciosissima troglodytes *[unpublished observations MB, TVN].

The present work investigates 13 populations representative of the two ecotypes, using sequencing of nuclear (*ITS2*) and mitochondrial (hereafter mtDNA) (*16S, COI *and *COII*) DNA regions as well as AFLP genome fingerprinting in a way to address the following questions:

1. Are ecotypes monophyletic?

2. Is adaptation to different habitats and host plants associated with genetic divergence?

## Results

### Phylogenetic reconstruction of the DNA sequence data sets

**Figure 2:**
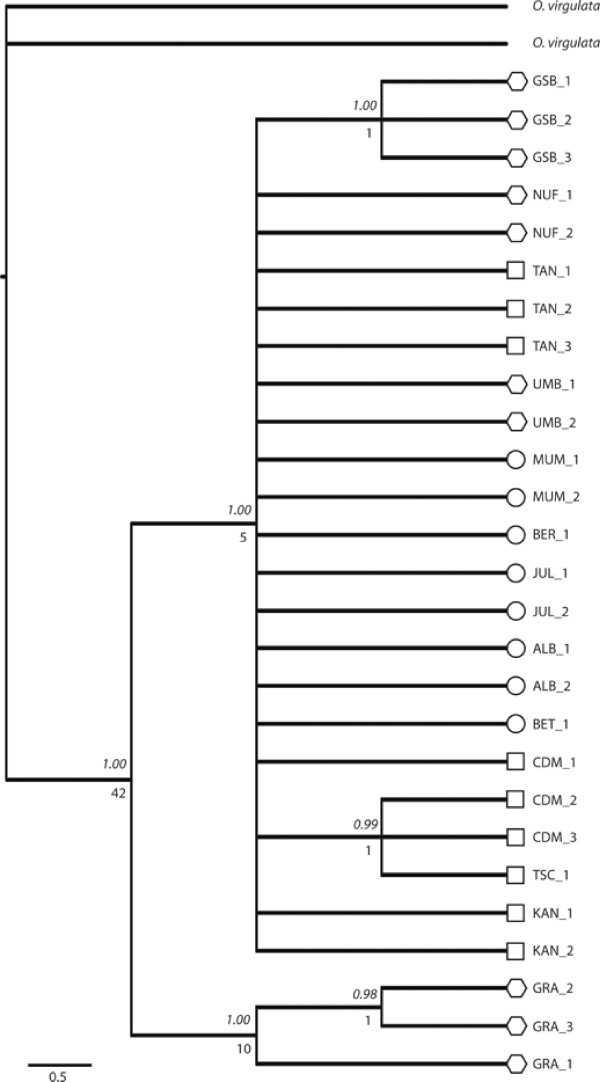
**Strict consensus tree of *Oreina speciosissima*, as revealed by mtDNA regions *16S, COI *and *COII *(maximum parsimony tree)**. Node supports are given by Bremer supports (decay index) ≥ 1 and Bayesian posterior probabilities (italic). Specimens are labeled according to morphotypes (i.e. square -*Oreina speciosissima sensu stricto, circle - Oreina speciosissima troglodytes *and polygon - intermediate forms).

The nuclear *ITS2 *region showed no variation for *Oreina speciosissima *and was thus excluded from further analyses. In contrast, the three mtDNA regions were polymorphic with a total alignment length of 1632 bp; 529 bp for *16S*, 470 for *COI *and 633 bp for *COII*. Excluding the outgroup, 30 characters were potentially parsimony informative (hereafter PPIc) among 37 variable characters. The three mtDNA regions contributed as follows: *16S *(3 PPIc among 5 polymorphic sites), *COI *(13 PPIc among 16 polymorphic sites) and *COII *(14 PPIc among 16 polymorphic sites). The best substitution models were Hasegawa-Kishino-Yano (HKY) [41] for *16S *and Hasegawa-Kishino-Yano plus Gamma (HKY+G) [41,42] for *COI *and *COII*. The alignments of the mtDNA markers were combined in a total evidence approach, after pairwise incongruence length difference ILD test [43] revealed no incongruence among the three mtDNA markers (*COI *and *COII, P *value = 1.00; *COI *and *16S, P *value = 1.00; *COII *and *16S, P *value = 1.00). The resulting dataset was investigated using maximum parsimony (hereafter MP) and Bayesian phylogenetic inference methods [44]. Both approaches produced highly congruent topologies with the same major nodes. The MP topology with Bremer supports [45] and corresponding Bayesian posterior probabilities from the Bayesian analysis (hereafter bpp) are shown in Figure 2. The ingroup is well supported with a Bremer support of 42 and a bpp of 1.00. The ingroup splits into two groups, a well-supported clade (Bremer support = 10 and bpp = 1.00) containing all individuals from the GRA population and a polytomy (Bremer support = 5 and bpp = 1.00) containing all other individuals. Apart from a clade containing all individuals of GSB and one with two individuals from CDM and one from TSC, there is no resolution within the polytomy. Sample NUF_3 failed to amplify and is therefore not shown in Figure 2. Only samples that rendered both satisfactory DNA sequences and AFLP fingerprints were used for phylogenetic analysis.

### AFLP

**Figure 3:**
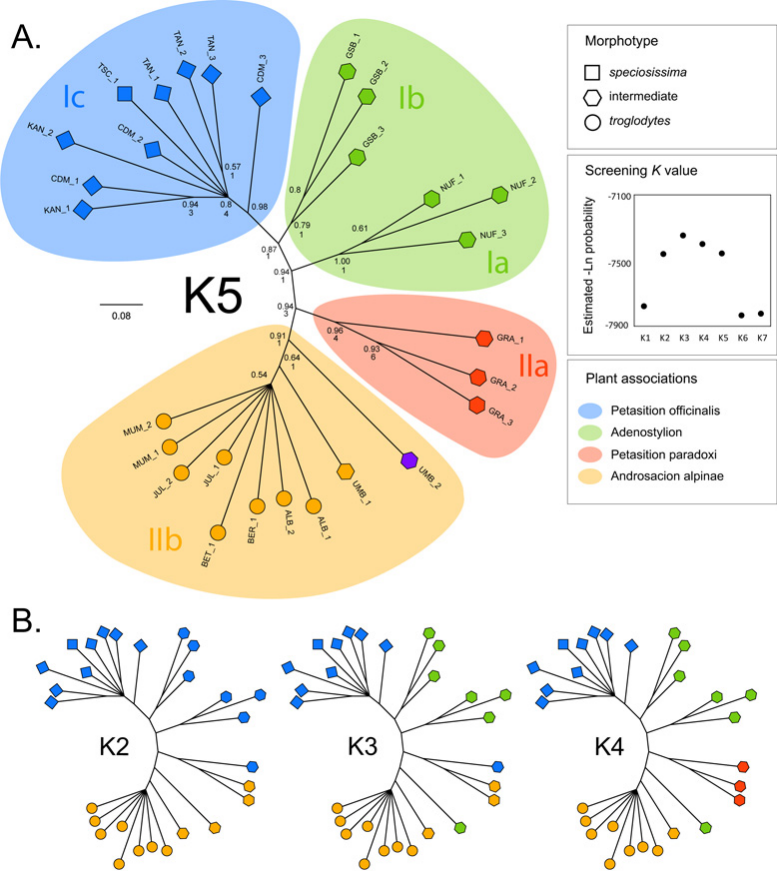
**Half-compat consensus tree and clustering of *Oreina speciosissima*, as revealed by AFLP data (Bayesian tree and STRUCTURE clustering)**. a) Specimens are labeled according to morphotypes (i.e. square - *Oreina speciosissima sensu stricto*, circle - *Oreina speciosissima troglodytes *and polygon -intermediate forms) and clusters defined using the Bayesian model-based STRUCTURE algorithm applied to AFLP data (i.e. colors of tips according to K = 5 groups; legend center panel - log-likelihood values of the best STRUCTURE runs for K1 to K7 groups, see text for further details). In addition, the corresponding habitat types (translated into plant associations) are displayed as color coded backgrounds. The names of clans (Ia,b,c and IIa,b, based on the Bayesian AFLP tree topology) and the node supports (i.e. above - Bayesian posterior probabilities and below - Bremer supports ≥ 1) are provided. b) Insights from alternative STRUCTURE results (i.e. K2 to K4). The tree and morphotype symbols are as in a).

The AFLP analysis produced a total of 530 bands (171, 166 and 173 for EcoRI-ACA/MseI-AGC, EcoRI-ACA/MseI-ACG and *E*coRI-ACA/MseI-AAC, respectively) with an average of 254 bands per individual and an average reproducibility rate of 96.1%. Among 510 variable characters, 458 were potentially parsimony informative. Just as the mtDNA data, the AFLP dataset was investigated using MP and Bayesian phylogenetic inference methods. Again, both approaches were highly congruent as the MP and Bayesian trees shared the same major nodes. Consequently, only the Bayesian phylogeny (including the bpp and Bremer supports) is displayed in Figure 3. Due to the lack of an outgroup, we present an unrooted topology (with supports extracted from the corresponding midpoint-rooted topology), which led to a separation of specimens into two well supported clans *sensu *Wilkinson *et al*. [46] (clan I and clan II), each with a bpp of 0.94 (Figure 3). Clan I includes three sub-clans supported with bpp values of 1.00 (Ia), 0.79 (Ib) and 0.98 (Ic) respectively. Clan I contains nine specimens with a strict *Oreina speciosissima sensu stricto *morphology (sub-clan Ic) and six specimens with an intermediate morphology (sub-clans Ia and Ib). Within clan II, two sub-clans were well supported with a bpp of 0.96 (IIa) and 0.91 (IIb) respectively. Clan II contains eight individuals with strict *Oreina speciosissima troglodytes *morphology (all of sub-clan IIb, except UMB specimens) and five individuals with an intermediate morphology (all of sub-clan IIa and UMB specimens from sub-clan IIb). Notably, specimens with intermediate morphologies were sorted close to the midpoint root of the tree topology. The AFLP dataset was further investigated using a Bayesian (i.e. STRUCTURE see [47,48]) and a distance-based (i.e. *K*-means; see [49,50]) clustering algorithm. The approaches produced fully congruent relationships and only results of the former are provided here. The STRUCTURE analysis showed highly likely clusters when considering *K *values ranging between two and five (see box Figure 3). The obtained results were largely congruent across *K *values (Figure 3) and highly compatible with the phylogenetic relationships. The only incongruence that could be observed when considering all *K *values, or when comparing STRUCTURE results with the tree topology, involved specimens with an intermediate morphology. Hereafter, we will consider and discuss results based on *K *= 5, given that they are the most informative. When viewed from a host plant perspective it becomes apparent that all leaf beetle specimens in clan II occur in the same stone run habitat with individuals from sub-clans IIa and IIb developing respectively in alkaline Petasition paradoxi and in acidic Androsacion alpinae habitats (Figures 1 and 3). In contrast all individuals from clan I occurred in high forbs, in Adenostylion or Petasites officinalis habitat, either on alkaline, neutral or slightly acidic soils [33] (see Figures 1 and 3).

## Discussion

### Are ecotypes monophyletic?

**Figure 4:**
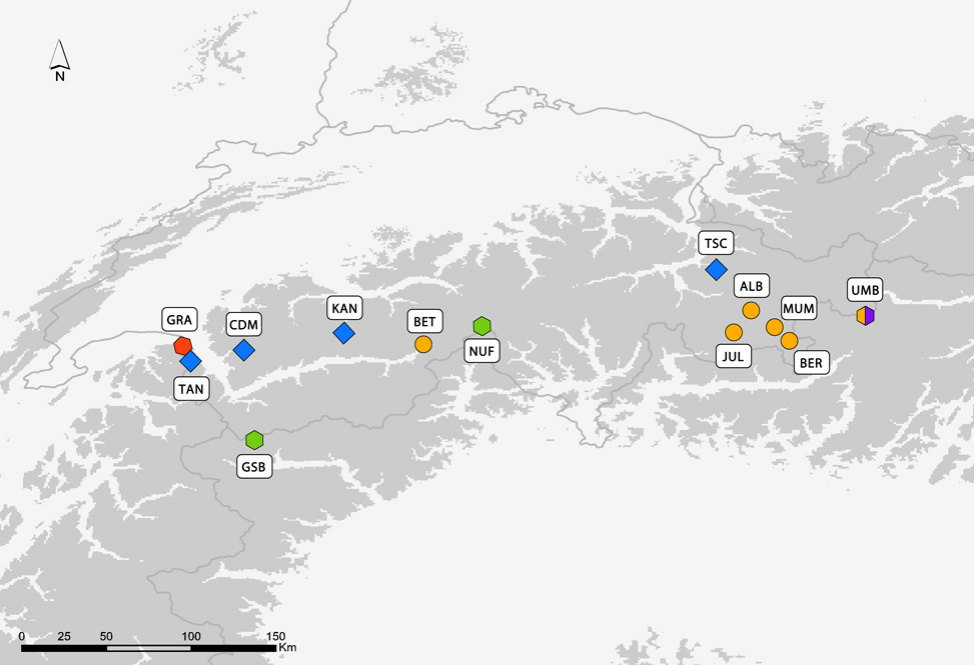
**Geographical distribution of the 13 sampled populations of *Oreina speciosissima *and results from AFLP Bayesian clustering**. Populations are labeled according to morphotype (i.e. square - *Oreina speciosissima sensu stricto*, circle - *Oreina speciosissima troglodytes *and polygon - intermediate forms) and clusters defined using the Bayesian model-based STRUCTURE algorithm (i.e. colors of tips according to K = 5 groups). Grey shaded areas represent elevated regions with altitudes above 1000 m. For full names of the populations see Table 1.

The phylogenetic tree based on mtDNA markers provides high support for the monophyly of *Oreina speciosissima sensu lato *(Figure 2). However, very little polymorphism and genetic structure are revealed within the ingroup, although the mtDNA markers proved variable enough to reconstruct intra-specific phylogenetic relationships in other arthropod systems (e.g [51-53]). Indeed, the resulting topology supports neither geographical nor ecotypic grouping of the beetles, possibly suggesting a recent divergence of *Oreina speciosissima *lineages, with the exception of specimens from the GRA population, which cluster as the sister lineage to all other specimens. Beetles from this latter population thus form an orphan clade [54], which may correspond to an isolated refugial lineage. AFLP data on the other hand shows a clear-cut differentiation of specimens (Figure 3). However, this pattern does not appear to have a geographical basis (Figure 4) and instead correlates with the beetle ecotypic definition, or in other words, to the plant habitat (Figure 1). In contrast, AFLP genetic structuring only partly correlates with morphotypes (sub-clans Ic and the larger part of IIb, see Figure 3). Ecotypes *per se *are thus not monophyletic, although there is a strong tendency for specimens and populations to cluster within the boundaries set by plant associations and their intrinsic ecologies.

### Is adaptation to different habitats and host plants associated with genetic divergence?

Our results showed that genetic differentiation among *Oreina speciosissima *lineages was clearly associated with plant communities (Figure 3). Accordingly, clustering in *Oreina speciosissima *is well explained by differences in bedrock type and host plants (translated here into different plant associations) (Figures 1 and 3). While specimens feeding in the Petasition paradoxi association (in which the calcicolous *Doronicum grandiflorum *is the main host plant for *Oreina speciosissima *[unpublished observations MB, TVN]) cluster in sub-clan IIa, specimens developing in the Androsacion alpinae association (in which the silicicolous *Doronicum clusii *is largely dominant as a host plant for *Oreina speciosissima *[unpublished observations MB, TVN]) are restricted to sub-clan IIb. The effect of soil acidity is less striking in clan I, probably because the Adenostylion and Petasition officinalis associations, which are characteristic of all specimens within this clan, are defined by intermediate soil pHs. These two plant communities include species showing an intermediate tolerance to acidic-alkaline variation, such as *Achillea macrophylla, Adenostyles alliarae *and *Petasites albus *[55]. Whereas the latter two represent the main host plant species of *Oreina speciosissima *in high forbs habitat [unpublished observations MB, TVN], other species (particularly in the Petasition paradoxi association) could play or have been playing the role of subalpine bridge species between the montane high forbs and alpine stone runs (see below). We are confident that these results are robust to potential shortcomings inherent to our limited sampling size (see [56] for a review). First, specimens were collected throughout the common geographical range of both ecotypes, a strategy that maximized both the phylogeographic and ecological representativity of our sampling. Second, robust and consistent results were obtained using both phylogenetic and clustering algorithms.

### Towards a scenario of ecological speciation in Alpine *Oreina speciosissima*

Although our data does not allow for divergence time estimates between *Oreina speciosissima *ecotypes, it seems likely that they diverged relatively recently. Indeed, the current distribution of *Oreina *populations suggest that the ecotype divergence might have arisen after one of the last glacial maxima, given that populations were probably not able to survive cold periods at high altitudes due to the presence of ice caps (with the possible exception of the GRA population; see above). This hypothesis is consistent with the low level of genetic variation observed in nuclear sequences and the low resolution in the mtDNA topology, as well as with a preliminary dating of the *Oreina *genus, in which the origin of *Oreina speciosissima *is estimated at circa 0.4 million years ago [36].

Our results suggest that from an ancestral niche associated with high forbs (see above) beetle populations were able to colonize new habitats along an altitudinal gradient (Figure 1) and invaded the acidic siliceous stone run habitat (corresponding to the Androsacion alpinae association), which is typical for Alpine regions in Central Europe. We propose that this habitat change could have been associated with host shifting events. Accordingly, the plant communities on which *Oreina *ecotypes feed appear to be connected by phylogenetically related host species. In a framework of plant-insect coevolution [57,58], adaptation to a given plant species might allow beetles to spread to other similarly-defended congeneric species [59,60]. Accordingly, *Doronicum *species occur in the Petasition paradoxi and the Androsacion alpinae, *Petasites *species link the Petasition officinalis to the Petasition paradoxi and finally, *Adenostyles *species are shared among the Adenostylion, the Petasition paradoxi and the Androsacion alpinae. Assuming host-plant conservatism, the connections described above might represent "shifting" routes that could explain how *Oreina speciosissima *lineages transited among habitats via host switching. Furthermore, these connections could account for the presence of putatively admixed specimens showing intermediate morphologies (e.g. UMB), thereby outlining a possible ongoing migration of beetles from one habitat to the other.

## Conclusions

Our study reveals a genetic structure in *Oreina speciosissima *as a function of the plant community in which beetles develop. We discussed several possible ecological features that could cause the divergence between ecotypes, among which the habitat and host-plant switches seem key factors. These results could be consistent with an ecological speciation scenario. Still, non-adaptive processes such as genetic drift, founder events and population bottlenecks might also have produced the observed pattern. Hence, further investigation is needed, for instance, fine scale studies relying on genomic approaches and targeting populations from a patchy distribution of the two ecotypes following an approach such as described by [61] could provide a powerful framework for detecting adaptive signatures associated to ecological speciation. Additionally reciprocal transplantation experiments in concert with crossings using local and non-local beetles could possibly reveal performance differences between locally adapted and non-adapted beetles and strengthen our argument for the existence of host races and ongoing or incomplete speciation (cf. [62,63]).

## Methods

### Sampling

During the summers of 2004, 2005 and 2008, specimens of *Oreina speciosissima sensu stricto *and *Oreina speciosissima troglodytes *were collected from 13 populations (Table 1) and stored in pure ethanol at -20°C. All sampled beetles were found on four distinct plant associations, namely Petasition officinalis, Adenostylion, Petasition paradoxi and Androsacion alpinae. The Petasition officinalis (populations CDM, KAN, TAN and TSC) and the Adenostylion (populations GSB and NUF) occurred on neutral to slightly calcareous bedrocks, at low and medium altitudes, respectively. The Petasition paradoxi (population GRA) and the Androsacion alpinae (populations ALB, BER, BET, JUL, UMB and MUM) grew on medium-high altitude calcareous and siliceous bedrocks, respectively (see Figure 1). Three individuals from each population were selected for genetic analysis, using only males to ensure accurate identifications based on genitalia. Following the reasoning of Nosil *et al*. 2002, 2003 [64,65] a 'population' is defined as all of the insects collected within a homogenous patch of plants belonging to one of the four abovementioned plant communities. 'Parapatric' populations are those in contact with a second population using host plants of a different plant community. If we take the maximum migration distance of *Oreina cacaliae *as reported in [40] as a proxy for migration ability of *Oreina speciosissima*, and thus the possibility for gene flow, this study incorporates only one true parapatric pair (TAN - GRA). As a result of this our study is not suitable to test the influence of geographical distance with regard to genetic distance between beetles that use different plant communities as host plants. The dataset was completed with two individuals of *Oreina virgulata *(i.e. a closely related species) that were used as the outgroup [34].

**Table 1:** Sampled populations of *Oreina speciosissima*

Code	Population	Altitude	Coordinates	Morphotype	Habitat	Year
KAN	Kandersteg	1314 m	46°28'21"N, 07°39'23"E	*speciosissima*	hf	2004
TSC	Tschiertschen	1325 m	46°48'55"N, 09°36'31"E	*speciosissima*	hf	2004
CDM	Col des Mosses	1716 m	46°23'26"N, 07°07'30"E	*speciosissima*	hf	2005
TAN	Lac Taney	1389 m	46°20'38"N, 06°50'01"E	*speciosissima*	hf	2008
GRA	Le Grammont	1974 m	46°21'15"N, 06°49'04"E	intermediate	sr	2008
NUF	Nufenenpass	2172 m	46°28'41"N, 08°22'36"E	intermediate	hf	2008
GSB	Grand St. Bernard	2410 m	45°52'04"N, 07°10'27"E	intermediate	hf	2008
BET	Bettmerhorn	2628 m	46°24'44"N, 08°04'33"E	*troglodytes*	sr	2008
UMB	Umbrailpass	2647 m	46°32'53"N, 10°25'43"E	intermediate	sr	2008
BER	Berninapass	2315 m	46°24'37"N, 10°01'36"E	*troglodytes*	sr	2008
ALB	Albulapass	2324 m	46°34'46"N, 09°50'15"E	*troglodytes*	sr	2008
MUM	Muottas Muragl	2735 m	46°30'27"N, 09°56'29"E	*troglodytes*	sr	2008
JUL	Julierpass	2373 m	46°28'02"N, 09°43'35"E	*troglodytes*	sr	2008

Sample locations with altitude (meters above sea level), coordinates (WGS 84) and year of collection with their codes, morphotype and habitat (hf: high forbs; sr: stone run).

### DNA sequence data and phylogenetic analyses

The DNA extraction, amplification and sequencing protocols as well as primers for the nuclear (*ITS2*) region and the three mtDNA markers (*16S, COI, COII*) are provided in [36]. The alignments of mtDNA markers (using the Clustal-Wallis algorithm [66]) were combined in a total evidence approach [67] after having performed pairwise incongruence length difference ILD tests [43]. We followed the snowball procedure as implemented in the program mILD[68].

Phylogenetic analyses were performed using the maximum parsimony (MP) and Bayesian Markov chain Monte Carlo (MCMC) criteria. Each partition and the combined data set were analyzed using parsimony ratchet [44] as implemented in PAUPrat[69] and further run in PAUP* 4b10 [70]. Ten independent searches were performed with 200 iterations and 15% of the parsimony informative characters perturbed [44]. The shortest most parsimonious trees were combined to produce a strict consensus tree. Branch supports were calculated using the Bremer support (also known as 'decay index') [45] as implemented in TreeRot[71] and further run in PAUP* 4b10 [70]. The Bremer support measures the number of extra steps in tree length required before a node collapses [45,72]. Model selection for the mtDNA data partitions in the MCMC was carried out with MrModeltest2 v.2.3 [73] based on the 'Akaike information criterion' [74]. Two Metropolis-coupled Markov chains with incremental heating temperature of 0.1 were run in MrBayes 3.1.2 [75] for 30 million generations and sampled every 1000^th ^generation. The simulation was repeated six times, starting from random trees. Convergence of the MCMC was checked using the Potential Scale Reduction Factor (PSRF) [76] implemented in MrBayes 3.1.2 [75] and the effective sample size (ESS) criterion for each parameter as implemented in Tracer 1.4 [77]. To yield a single hypothesis of the phylogeny, the posterior distribution was summarized in a 50% majority rule consensus tree (the "halfcompat consensus tree" from MrBayes) after burn-in (for each analysis 10000 trees were discarded). The combined dataset was analysed using partition specific model parameters [73].

### AFLP

Genome fingerprinting was performed using the AFLP protocol described in [78]. The selective amplifications were performed using 5-FAM fluorescently labelled *Eco*RI primer (i.e. *Eco*RI + ACA) with one of the following: MseI primer + AXX (AGC, ACG and AAC). All amplifications were run in a Biometra TGradient thermocycler (Biometra, Göttingen, Germany). Samples were randomly displayed on a 96-well PCR plate, with ten individuals being replicated to assess the overall reproducibility of reactions. PCR products were analysed using the GeneScan technology with a capillary sequencer (ABI 3730XL, Applied Biosystems, Foster City, CA; the service was provided by Macrogen Inc. Seoul, South Korea).

Resulting electropherograms were analysed with PeakScanner (ABI, peak detection parameters: default parameters with the addition of a light peak smoothing) in order to detect and calculate the size of AFLP bands. The scoring was performed using an automated scoring R CRAN package, RawGeno 2.0 [79,80]. The library was settled as follows: scoring range = 100 - 250 bp for EcoRI-ACA/MseI-AGC, EcoRI- ACA/MseI-ACG and 100-280 for *E*coRI-ACA/MseI-AAC, minimum intensity = 50 rfu, minimum bin width = 0, maximum bin width = 1 bp and closely sized bins (5%) were removed. Finally, the matrices of the three scored primer pairs were concatenated into a single binary matrix where individuals and bands were stored as lines and columns, respectively.

### Phylogenetic and clustering analyses of the AFLP data set

Phylogenetic analyses of the AFLP data were performed using the MP and Bayesian MCMC criteria. The MP analysis (including Bremer support analysis) was performed as described above. Parameters for the Bayesian MCMC analysis performed in MrBayes 3.1.2 were set as follows: "datatype = restriction" and "coding = noabsencesites". Four metropolis-coupled Markov chains with incremental heating temperature of 0.1 were run for 5 million generations and sampled every 1000^th ^generation. The simulation was repeated six times, starting from random trees. Convergence of the analysis was checked using the PSRF and ESS criteria (see above for more details). The posterior distribution was summarized in a halfcompat consensus tree (see above) after burn-in (for each analysis 1500 trees were discarded).

Two independent clustering algorithms were used to assign *Oreina speciosissima *specimens into a user-defined number of groups (hereafter *K*). First, we used non-hierarchical *K*-means clustering [81], a distance-based algorithm that proves reliable in an AFLP framework [49,50,82]. A total of 100 000 independent runs was carried out for each value of *K *clusters assumed (i.e. ranging from two to seven) and only runs yielding the highest inter-cluster variance were considered for further analysis. The optimal *K *value was determined based on the second derivative of the intercluster inertia, as in [50]. Computations were performed using R CRAN [83] (script available upon request to NAR). Second, we performed a model-based Bayesian inference clustering as implemented in STRUCTURE 2.2 [47,48]. The analysis assumed an admixture model and independent allele frequencies between clusters. Five independent runs were carried out for each value of *K *(i.e. ranging from one to seven), with parameters and model likelihood estimated over 1 000 000 MCMC generations (following a burn-in period of 200 000 generations). For each *K *value, only runs that obtained the highest likelihood value were taken into account for further analyses. The majority-rule criterion (>0.5 in the assignment probability) was applied to assign samples to a given cluster as in [50]. Both clustering approaches provided fully congruent insights and therefore only results from STRUCTURE are displayed here.

## Authors' contributions

MB and TVN collected the samples, carried out the morphological and genetic analyses, participated in phylogenetic analysis and drafted the manuscript. NAR, SB and NAL designed phylogenetic tools, participated in phylogenetic analysis and revised the manuscript. All authors read and approved the final manuscript.
